# Global trends in research on oxidative stress associated with periodontitis from 1987 to 2022: A bibliometric analysis

**DOI:** 10.3389/fimmu.2022.979675

**Published:** 2022-09-08

**Authors:** Xirui Xin, Xingchen Xiang, Yu Xin, Qiong Li, Haonan Ma, Xinchan Liu, Yubo Hou, Weixian Yu

**Affiliations:** ^1^ Department of Periodontology, Hospital of Stomatology, Jilin University, Changchun, China; ^2^ Jilin Provincial Key Laboratory of Tooth Development and Bone Remodeling, Hospital of Stomatology, Jilin University, Changchun, China; ^3^ Department of Oral Implantology, Hospital of Stomatology, Jilin University, Changchun, China

**Keywords:** oxidative stress, periodontitis, inflammation, bibliometric analysis, data visualization

## Abstract

**Background:**

Oxidative stress has been implicated in many chronic inflammatory diseases, including periodontitis. To date, however, only a few bibliometric analyses have systematically studied this field. This work sought to visualize research hot spots and trends in oxidative stress associated with periodontitis from 1987 to 2022 through bibliometric approaches.

**Methods:**

The Web of Science Core Collection was searched to retrieve relevant publications. HistCite, VOSviewer, and CiteSpace were used to perform bibliometric analysis visually in terms of annual output, active countries, prolific institutions, authors, core journals, co-cited references, and co-occurrence of keywords.

**Results:**

A total of 1654 documents were selected for analysis. From 1 January 1987 to 11 June 2022, the number of annual publications related to oxidative stress in periodontitis exhibited an upward trend. The most prolific country was China with 322 documents, but the United States had 11334 citations. Okayama University, University of Birmingham, and Sichuan University were the most active and contributive institutions. The Journal of Periodontology ranked first in terms of numbers of publications and citations. Ekuni was the most prolific author, while Chapple ranked first among co-cited authors. The Role of Reactive Oxygen and Antioxidant Species in Periodontal Tissue Destruction published by Chapple was the most frequently co-cited reference. Keywords co-occurrence showed that oxidative stress was closely related to inflammation, antioxidants, and diabetes.

**Conclusion:**

Our research found that global publications regarding research on oxidative stress associated with periodontitis increased dramatically and were expected to continue increasing. Inflammation and oxidative stress, and the relationship between periodontitis and systemic diseases, are topics worthy of attention.

## Introduction

Periodontitis is a chronic progressive inflammatory disease that can destroy the supporting apparatus of the teeth and subsequently lead to tooth loss (1). An estimated 740 million people worldwide are affected by the severe forms of this disease (2). Numerous experimental studies have shown a strong association between oxidative stress and periodontitis (3, 4). As periodontitis progresses, excessive reactive oxygen species (ROS), mostly produced by overactive neutrophils, can exert cytotoxic effects and not only interfere with cell growth and the cell cycle process but also induce apoptosis of gingival fibroblasts, resulting in serious damage to the periodontal tissue (5, 6). Evidence that illustrates the role of ROS in establishing an oxidatively stressed environment that underlies the pathogenesis of a wide range of chronic inflammatory conditions, such as type 2 diabetes mellitus, inflammatory bowel disease, Alzheimer’s disease, and periodontitis (7), has been increasing significantly. In addition, Sczepanik et al. indicated that the upregulation of ROS might play one of the most critical roles in the establishment and progression of inflammatory diseases (8). Collectively, oxidative stress is closely related to inflammatory response and may be the key target of many inflammatory diseases, including periodontitis.

Oxidative stress is caused by ROS (9). ROS are highly active molecules that include at least one unpaired electron (particularly superoxide ${\text{O}}_{2}^{-}\cdot$ , hydroxyl radical •OH, and hydrogen peroxide H_2_O_2_). Since the “free radical theory” was proposed in the 1950s and revised in the 1970s, oxidative damages have been believed to play a pivotal role in pathological processes (10). Periodontitis is characterized by chronic inflammation associated with alteration of the oral microbiota (11). Microbial dysbiosis that occurs in periodontal disease results from a hyperinflammatory state in the host (12). Certain species ultimately become dominant as dysbiosis progresses (13). Many pathogenic bacteria, such as *Porphyromonas gingivalis* and *Tannerella forsythia*, play a vital role in the development of periodontitis (14, 15). Excessive inflammation in response to bacterial plaque leads to the release of ROS from neutrophils. However, although the primary function of ROS is to kill bacteria, excessive ROS can promote tissue damage and cause an intense inflammatory reaction (5, 16). Therefore, maintaining the balance of ROS plays a key role in the development of periodontal pathology. Balance exists between ROS and antioxidants under normal physiological conditions. Oxidative stress only occurs when the antioxidant defense system cannot neutralize the increased amount of ROS (17, 18). Consequently, promising therapies to prevent the formation of oxidative stress or improve the capacity of the antioxidant system have been proposed. Some clinical studies have shown that antioxidants can remarkably alleviate the inflammatory progression of periodontitis and effectively improve the clinical indications related to periodontitis, such as the depth of periodontal pockets and the loss of attachment (19, 20). Marconcini et al. (21) further showed that periodontal antioxidants treatment can effectively reduce plasma reactive oxygen metabolites in patients with diabetes, which indicates that antioxidants are an effective strategy for the treatment of periodontitis, and that the treatment of periodontal disease may help control blood glucose and reduce systemic inflammation. Thus, exploring the application of antioxidant treatment strategies may be of high significance not only for periodontal disease but also for various systemic diseases.

Bibliometrics, which was proposed by Alan Pritchard in 1969, can conduct retrospective reviews, look for data correlations, and predict future developments (22). Bibliometric studies are widely used to gauge the scholarly impact of scientific publications and are used as an important vehicle for highlighting and motivating emerging scholarship. (23, 24). Bibliometrics and visual analysis cannot only improve the understanding of research activities by effectively integrating information, but they also help scholars quickly grasp research hot spots and future trends in a specific field (25). Over the years, many bibliometrics have been applied to the medical fields of gynecology, orthopedics, complementary and alternative medicine, which has extensively promoted the development of medical research and clinical practice (26). However, bibliometrics is still rarely applied in the field of stomatology, and the study on oxidative stress related to periodontitis remains a void. Thus, the current work aims to determine and study the characteristics of documents about oxidative stress associated with periodontitis, summarize current achievements in this field, evaluate the academic influence and characteristics of publications in this field, and provide new design ideas for further investigation.

## Materials and methods

### Data source and search strategy

**Figure 1:**
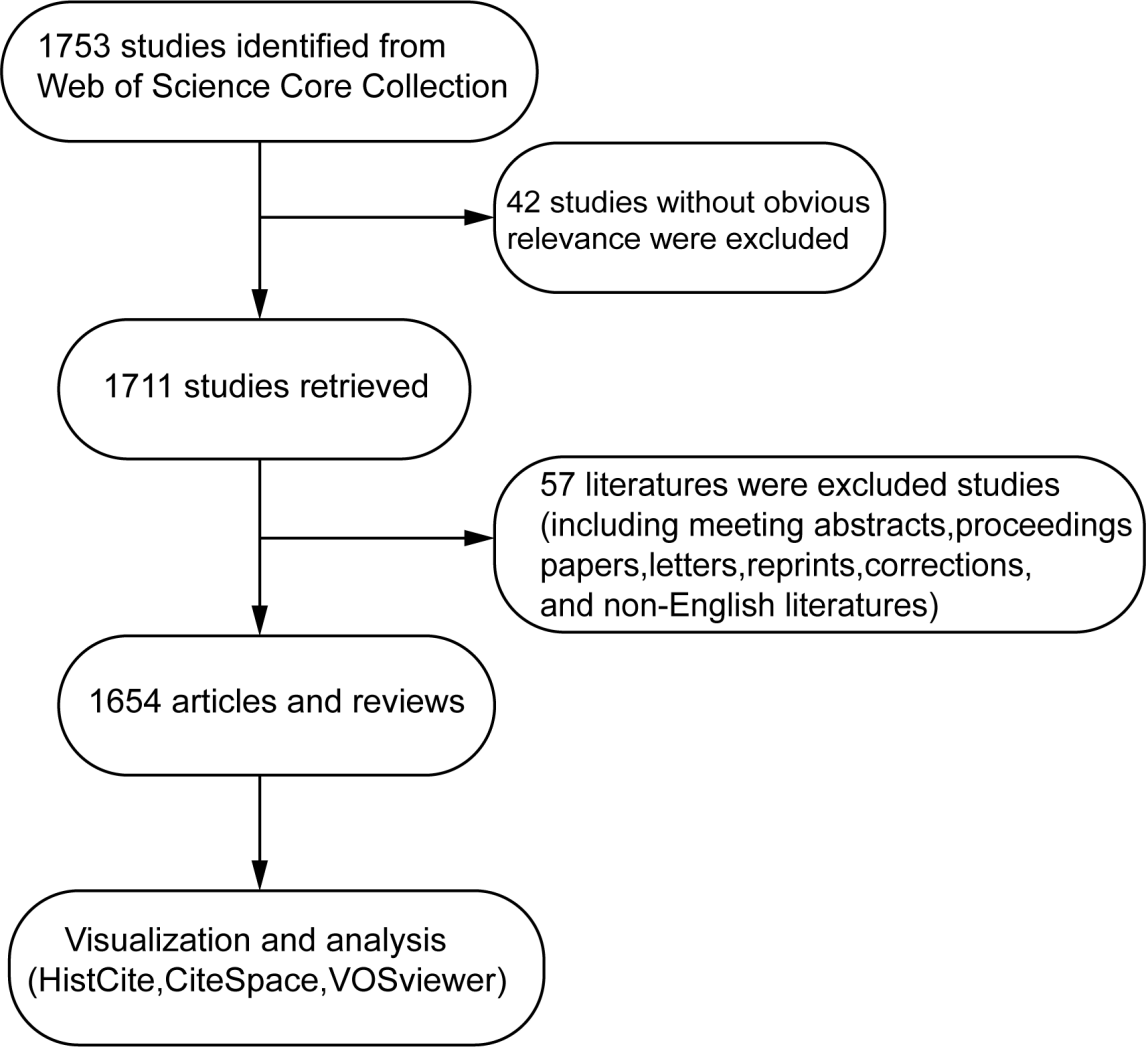
Flowchart of our study.

Related publications were collected from the Web of Science Core Collection (WOSCC) and limited from 1987 to 2022. We conducted relevant pre-tests and optimized the retrieval strategy to ensure the integrity and accuracy of the results. The search terms and strategies used were as follows: TS = (“oxidative stress” OR “ros” OR “reactive oxygen species”) AND TS = (periodontal* OR periodontitis). Since 1987, the first article that meets the inclusion criteria has been included. Considering the rapid update of the database, the literature search is carried out in one day. Articles and reviews occupy the main part of the database, which can represent the trend of the whole research field. In addition, to minimize the omission of information, we also use a series of recursive searches. In terms of language, English is a universal language in the world, so we consider that English literature is more standard and meaningful than other languages. In addition, the search strategy is based on the machine algorithm of the database, so the literature that has not been published by the publishing institution and without complete information cannot be extracted. Based on these considerations, documents that meet the following criteria were included. (1) Paper was published from 1 January 1987 to 11 June 2022. (2) The literature type is “ARTICLE” or “REVIEW.” (3) The literature reports a research on oxidative stress and periodontitis. (4) The language is English. The following documents were excluded. (1) The type of literature is a meeting abstract, an editorial material, a correction, a letter, a proceeding paper, or a repeated article. (2) The literature type has no significant correlation with periodontitis or oxidative stress. (3) The language is not English. (4) Unpublished documents without sufficient information. The workflow of this study is presented in **Figure 1**. The “full records and cited references” function of the Web of Science was used to export the data, all valid data saved as raw text files and stored in the format of download_txt for further analysis. Studies that met the inclusion criteria were independently reviewed by two researchers (XRX and XCX), who extracted and analyzed the following relevant literature measurement indicators: basic information (number of articles, total citations, average citations of each article, and annual publications and citations), top 10 prolific countries/regions and institutions, top 10 productive journals and co-cited journals, top 10 active and co-cited authors, top 10 co-cited references, and co-occurrence of keywords. Any disagreements between the two researchers (XRX and XCX) were resolved by negotiation, and, if not reached, an experienced expert (WXY) in this field was consulted to resolve the issue.

### Analytical methods

Bibliometrics first appeared in the early 1900s and formed an independent discipline in 1969 (27). On one hand, the bibliometric analysis provides a quantitative analysis method for reviewing and observing the literature in a specific field (28). Details such as countries, institutions, literature, authors, keywords, etc. can be obtained in the process of analysis. On the other hand, the emergence of visual graphics can enhance the effect of document analysis with the help of modern computer technology. In particular, with the use of visual analysis tools, the readability of data is enhanced and the results are more comprehensive now. Visualization emphasizes the connection between information, and explores the correlation between different authors, institutions, and countries in the same research field. Visualization is also helpful in exploring the internal relationship of various information, such as different authors having the same research field, the direction of research and focus of different institutions, the new theories of existing institutions and so on (29).

HistCite (version 12.03.17), CiteSpace (version 5.8.R3), and VOSviewer (version 1.6.18) were used in data analysis and visualization, including collaborative networks of countries/regions, institutions, and co-occurrence of keywords. Co-citation analysis of authors and references was also performed. HistCite, developed by Eugene Garfield, is a powerful citation analysis software for drawing the development background of a research field and performing direct citation linkages between scientific papers (30). HistCite is free and easy-to-use software program. After importing the initial document exported from the Web of Science into the txt folder that is specified by the software, then the software is started which can automatically read the information contained in the form of raw data and then present it in the form of web pages, mainly including yearly output, active countries, top institutions, core journals, and active authors. Countries, institutions, or authors will rank automatically according to the number of documents. The ranking information can be exported to Microsoft Excel 2016 or to other software when necessary.

CiteSpace, a software developed by Chaomei Chen, can visualize the network among collaborations, document citations, and research hotspots (31). In our study, the details on CiteSpace settings were as follows: Time slicing was performed from 1987 to 2022, for 5 year per slice. Term source (title, abstract, author keywords, keywords plus) and links (strength: cosine, scope: within slices) were set normally. The top 50 most frequently cited or occurring items were selected. The Pathfinder was used to prune unnecessary links.

VOSviewer, developed by the Center for Science and Technology Studies at Leiden University, is a program for constructing and viewing bibliometric maps. It can be used to create author or journal maps based on collaborative data or build keyword maps based on co-occurrence data (32). In this study, the parameters of the VOSviewer were as follows: The counting method was selected for “full counting.” The minimum number of citations for the co-cited authors and co-cited references was twenty and forty, respectively. The unit of analysis of the co-occurrence keyword was “all keyword,” and the threshold for the minimum number of occurrences was set to thirty. The form of the visualization diagram was network visualization for the co-cited authors and occurrence keywords. The label was set to circles, and the keywords or authors appeared as the nodes. Besides, the co-cited references were generated as the form of density visualization, and the rainbow color was chosen to highlight the area of high density.

These analytical tools provide objective and diverse perspectives on the role and development of oxidative stress in periodontitis.

## Results

### General statistics

**Figure 2:**
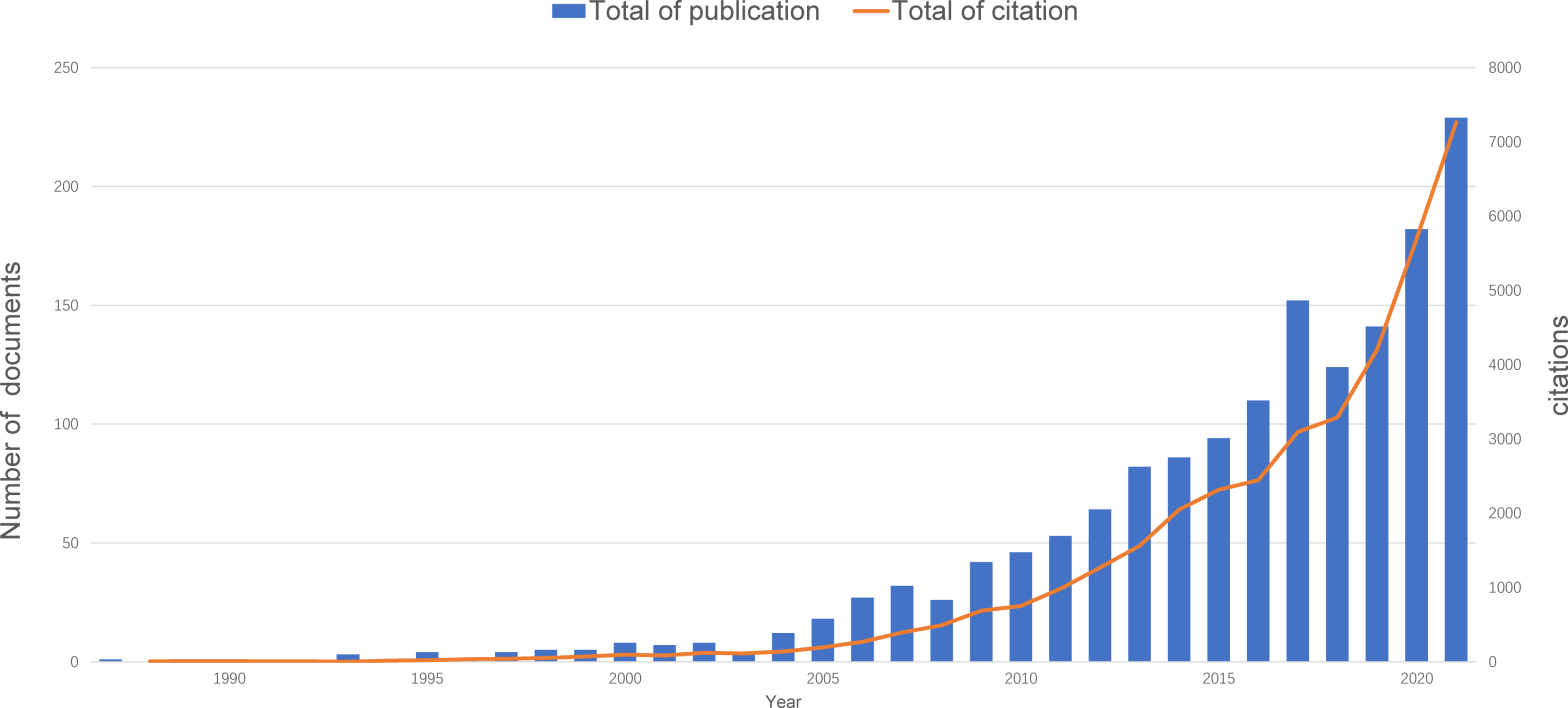
Trends in the annual number of publications and citations.

According to our search criteria, a total of 1654 publications were obtained from WOSCC for the period of 1987–2022, including 1395 articles(84.3%) and 259 reviews(15.7%). To date, all the publications have been cited for a total of 39,666 times, and the average number of citations per article is 23.9 times. These publications came from 71 countries/regions, 1630 institutions, 6888 authors, and 578 journals. As shown in **Figure 2**, the annual publication output generally exhibited a rapid growth trend beginning in 2004. Moreover, the number of citations of these publications also sustained an annual growth after 2004.

The results suggested that this field developed rapidly in the past two decades, and the number of papers published in recent years was still within a period of rapid growth, indicating that a growing number of scholars are continuing to give attention to this field and conduct relevant studies.

### Analysis of countries/regions and institutions

**Figure 3:**
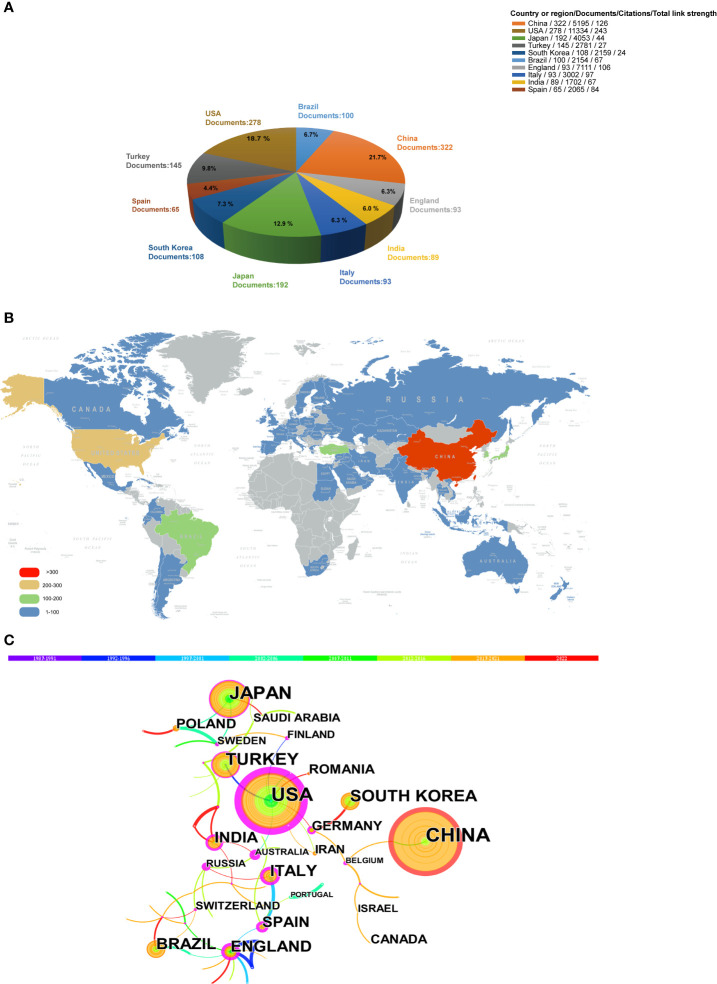
Distribution of publications from different countries/regions: **(A)** Top 10 productive countries/regions in this field. **(B)** spatial distribution of global publications and **(C)** collaboration analysis of countries/regions.

We ranked 10 high-output countries/regions in accordance with the number of publications. As shown in **Figure 3A**, China, the United States, and Japan made outstanding contributions to the development of publications on oxidative stress in periodontitis. **Figure 3B** shows the global distribution of publications in this field. China published more than 300 publications, followed by the United States (between 200 and 300). Four countries, namely, Japan, South Korea, Brazil, and Turkey, have a number of publications ranging from 100 to 300. Most countries/regions have published less than 100 articles. **Figure 3C** illustrates the cooperative relationship between different countries/regions. The nodes represent countries/regions, while the lines between the nodes represent cooperation relationships. The larger the node size, the higher the number of publications. Different colors inside the nodes represent various time intervals. The most representative is China, with the color rings inside the nodes indicating that its publications are concentrated in recent years. The nodes in the outermost region with purple rings represent high centrality. Some countries or regions are located at the important connection points of key nodes; hence, their centrality is stronger, and the corresponding purple rings at the outermost circle of nodes are larger. Thus, the United States, Australia, England, and Italy exhibited strong centrality, i.e., they were in a relatively central position in the global cooperation network and cooperated closely with many countries or regions. Although China had a large number of documents, it exhibited no advantage in centrality and had not established extensive cooperation with other countries.

**Figure 4:**
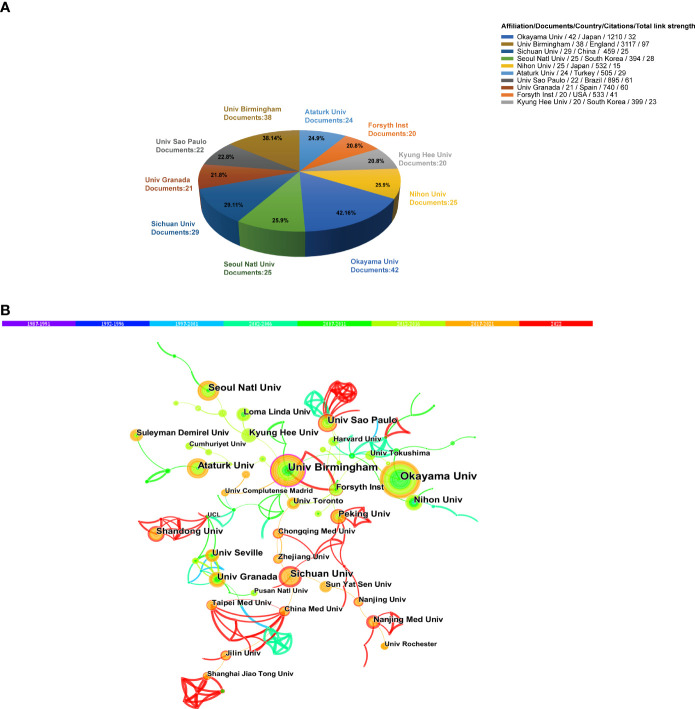
**(A)** Top 10 productive institutions related to oxidative stress in periodontitis research. **(B)** Collaboration analysis of institutions.

The 10 most productive institutions are shown in **Figure 4A**. The leading institutions are Okayama University (Japan), the University of Birmingham (England), and Sichuan University (China). Although the University of Birmingham had the second largest number of documents compared with Okayama University, it exhibited high centrality in the cooperation network among different institutions (**Figure 4B**). Moreover, all the top 10 institutions were from countries with highly productive publications.

### Analysis of journals and co-cited journals

**Figure 5:**
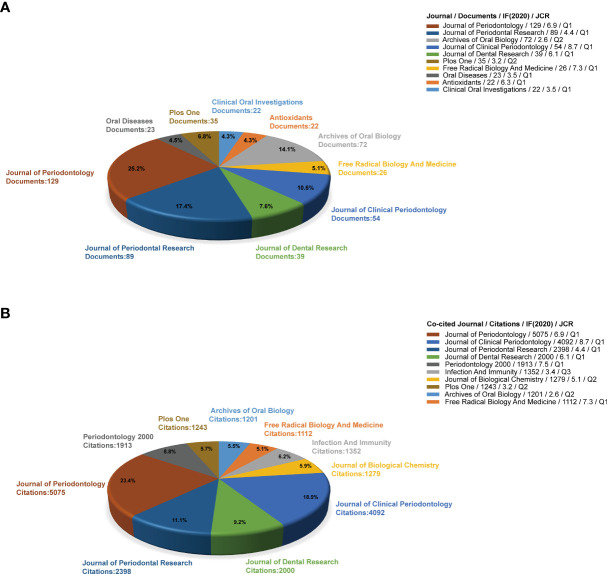
**(A)** Top 10 productive journals related to oxidative stress in periodontitis research. **(B)** Top 10 co-cited journals related to oxidative stress in periodontitis research.

The 10 most productive and co-cited journals are shown in **Figures 5A**, **B**, respectively. Approximately 30% of the papers were published in the top 10 academic journals (511/1654). The Journal of Periodontology (129 publications, impact factor [IF]: 6.9) published the most papers about oxidative stress in periodontitis research, while the Journal of Periodontal Research (89, IF: 4.4) and the Archives of Oral Biology (72, IF: 2.6) ranked second and third, respectively. The Journal of Clinical Periodontology had the highest IF (8.7) in 2020 among the top 10 productive journals. From the partition analysis of Journal Citation Reports, 8 of the top 10 most prolific journals were classified under Q1, while the remaining two were classified under Q2. The Top 5 co-cited journals related to oxidative stress in periodontitis research were all classified under Q1. Interestingly, the Journal of Periodontology had the highest output and was the most frequently co-cited. It may also be the most popular journal in this field. Moreover, high-productivity and highly co-cited journals mostly overlapped, i.e., seven journals were the same. The top five journals and co-cited journals were all dental professional journals. Furthermore, the top three co-cited journals were all periodontal-related professional journals.

### Analysis of authors and co-cited authors

**Figure 6:**
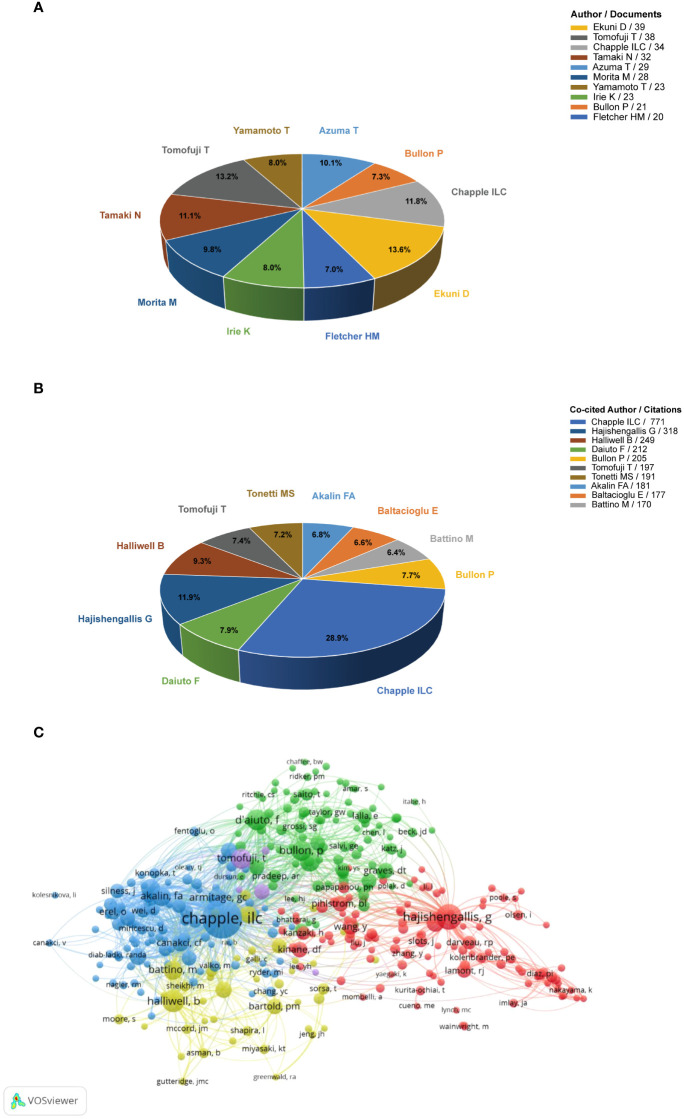
**(A)** Top 10 authors with the most publications in this field. **(B)** Top 10 co-cited authors with high citations in this field. **(C)** VOSviewer network visualization map of co-cited authors.

The top 10 authors in terms of the number of publications and highly co-cited authors are shown in **Figures 6A**, **B**. The top 10 productive authors contributed 287 publications, accounting for 17.4% (287/1654) of the total number of papers. Ekuni (39 publications) published the most number of publications, followed by Tomofuji (38 publications) and Chapple (34 publications). The network visualization map of co-cited authors is shown in **Figure 6C**. The largest nodes were associated with the most frequently co-cited authors. The top three co-cited authors were Chapple (771 citations), Hajishengallis (318 citations), and Halliwell (249 citations). The network visualization map of co-cited authors shows that citations are broadly linked, indicating that the links between studies are relatively close.

### Analysis of co-cited references

**Figure 7:**
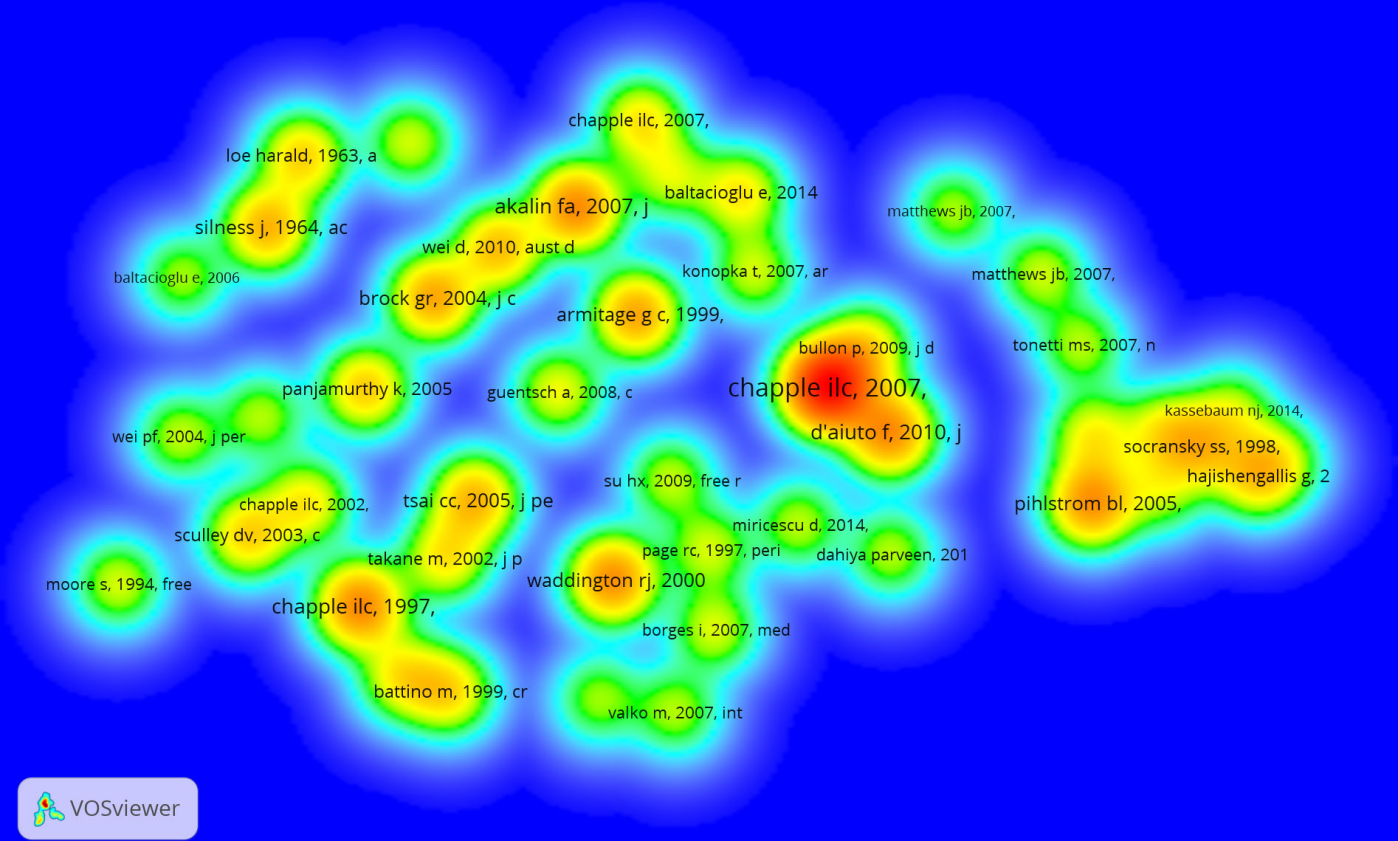
VOSviewer visualization map of co-cited references.

The top 10 most co-cited references included 5 reviews and 5 research articles (**Table 1**). The top two references with the most co-citations were all from Chapple. His two publications were both reviews, with the frequency of co-citation being 251 and 129, respectively, followed by Akalin, with the frequency of co-citation being 127. Through the analysis of VOSviewer software, the co-citation visualization map of references is shown in **Figure 7**. Each reference in density visualization has colors that indicate the frequency of co-citation. Red means a high frequency of co-citation, while green denotes a low frequency of co-citation. In addition, these references are distributed at different distances from one another. The closer their distance in space, the closer the relationship between articles. Notably, Chapple appears four times in **Figure 7**, once in 1997, once in 2002, and two times in 2007, further indicating that the author made outstanding contributions to this research field. His studies were widely cited by researchers. This finding also coincided with the analysis of the co-cited authors mentioned earlier.

**Table 1:** Top 10 co-cited references related to oxidative stress in periodontitis.

Rank	Title	Type	First author	Source	Publication year	Co-citations
1	The role of reactive oxygen and antioxidant species in periodontal tissue destruction	Review	Chapple ILC	Periodontology 2000	2007	251
2	Reactive oxygen species and antioxidants in inflammatory diseases	Review	Chapple ILC	Journal of Clinical Periodontology	1997	129
3	Lipid peroxidation levels and total oxidant status in serum, saliva and gingival crevicular fluid in patients with chronic periodontitis	Article	Akalin FA	Journal of Clinical Periodontology	2007	127
4	Oxidative Stress, Systemic Inflammation, and Severe Periodontitis	Article	Daiuto F	Journal of Dental Research	2010	124
5	Periodontal disease mechanisms - Reactive oxygen species: a potential role in the pathogenesis of periodontal diseases	Review	Waddington RJ	Oral Diseases	2000	123
6	Periodontal diseases	Review	Pihlstrom BL	Lancet	2005	120
7	Development of a classification system for periodontal diseases and conditions.	Review	Armitage GC	Annals of Periodontology	1999	107
8	Local and systemic total antioxidant capacity in periodontitis and health	Article	Brock GR	Journal of Clinical Periodontology	2004	106
9	Periodontal Disease in Pregnancy II. Correlation Between Oral Hygiene and Periodontal Condition.	Article	Silness John	Acta odontologica Scandinavica	1964	98
10	Lipid peroxidation: a possible role in the induction and progression of chronic periodontitis	Article	Tsai CC	Journal of Periodontal Research	2005	96

### Analysis of co-occurrence keywords

**Figure 8:**
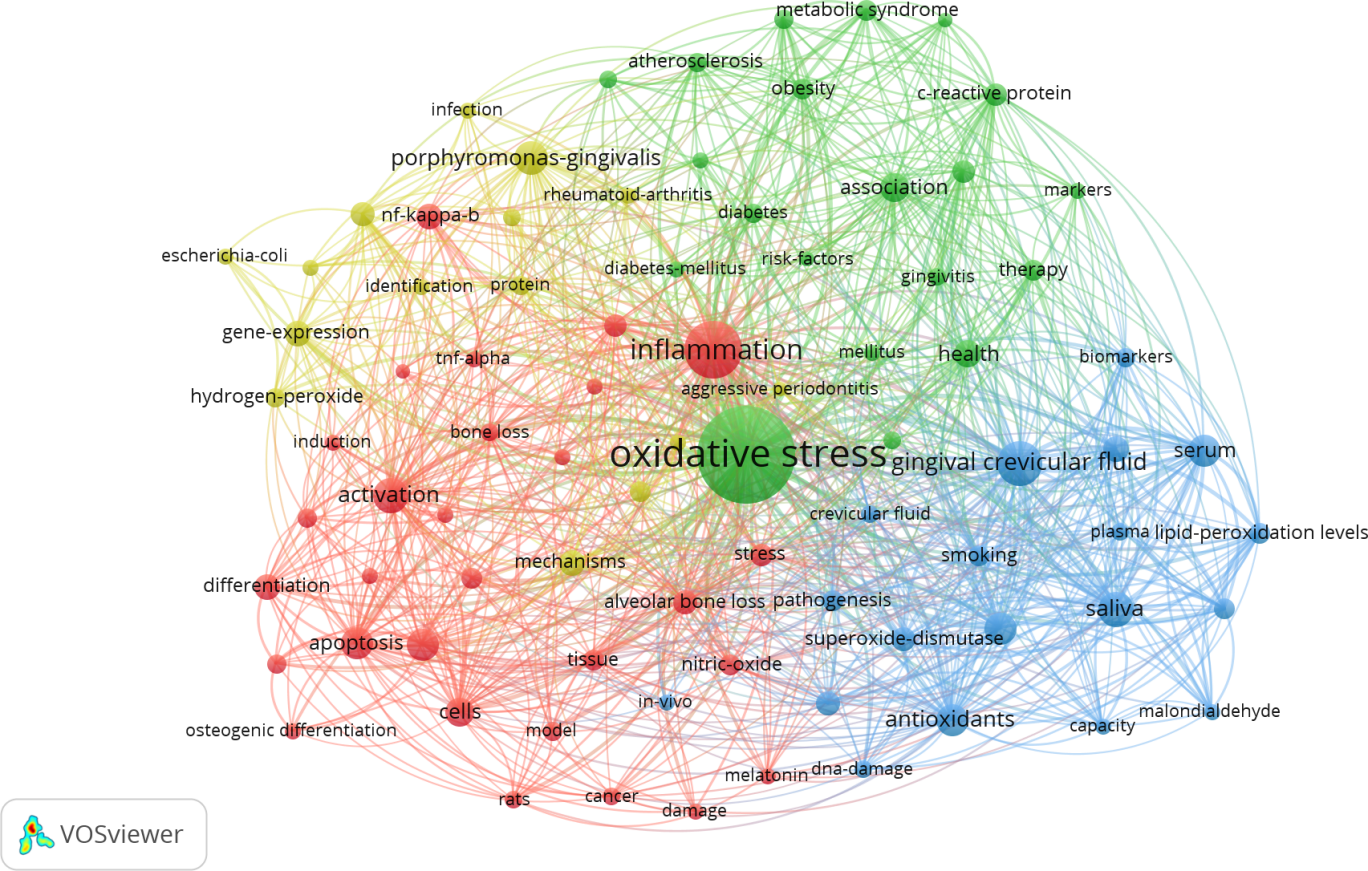
VOSviewer visualization map of co-occurrence keywords.

VOSviewer was used to generate a keyword co-occurrence map. After deleting invalid and duplicated keywords, the collaboration network was obtained in **Figure 8**. Weight is determined by the frequency of keywords. Therefore, the larger the node, the higher the frequency of these keywords in all the papers. Based on the analysis of the software visualization results, keywords, such as oxidative stress, inflammation, antioxidants, and diabetes, exhibited a high frequency in these publications. Moreover, nodes and labels form a unit, and units of varying colors form different clusters. Red, green, blue, and yellow clusters represent four different research directions. The keywords for the green cluster were diabetes, obesity, atherosclerosis, and metabolic syndrome. The keywords for the red cluster were inflammation, Nf-κB, TNF-α, and apoptosis. The keywords for the yellow cluster were *Porphyromonas gingivalis*, *Escherichia coli*, and infection. The keywords for the blue cluster were gingival crevicular fluid, serum, biomarkers, and antioxidants.

## Discussion

In this study, we performed a bibliometric analysis to assess the development trends and hotspots of research on related fields of oxidative stress and periodontitis. We retrieved 1,654 original articles and reviews published from 1987 to 2022. During the research period, only a few papers were published in this field before 2004. Thereafter, a period of rapid development was ushered in. The most productive period was 2021, indicating the rapid growth and continuous research interests in this field. Thus, we can speculate that research on oxidative stress associated with periodontitis will remain a hot spot in the future.

With regard to countries/regions, the analysis results indicated that the number of global publications was unbalanced among countries and regions, and only 6 countries had published more than 100 documents. Although China led in the number of publications, the citation of its publications was less than that of others. For example, China had more publications than the United States, but the United States was about two times as large as China in terms of citations and total link strength (**Figure 3A**), probably because publications from China mostly appeared in recent years. Notably, England ranked seven in the number of publications, but its publications had been cited more than those of China. In according with the visual network diagram (**Figure 3C**), England might have benefited from the advantages of conducting research earlier and its close ties with other countries in the global cooperation network. Therefore, countries and regions should remove academic barriers and strengthen cooperation and exchanges with one another. Transnational and cross-team cooperation is the key to accelerating the global development of disciplines. In addition, there are many factors that may affect the uneven number of documents published by countries/regions. Citation counts can be used as an indicator of research quality (33). Many authors believe that publishing their article in a journal of high impact factors will improve the citation of their paper. In fact, there is no correlation between journal impact factors (JIF) and the frequency with which an article is cited (34, 35). Nevertheless, the JIF continues to be used as a surrogate measure of scientific quality in many countries (36). In recent years, funding agencies and governments usually use JIF based on a two-year citation window to evaluate and compare the scientific performance of individuals, research groups and institutions (37). Therefore, JIF has a potential impact on the number of publications and citations of different countries/regions, and it is worth studying and paying attention to.

In terms of institutions, Okayama University and the University of Birmingham contributed to the research front. In particular, the University of Birmingham in the United Kingdom, exhibited extensive cooperation and took the lead in total link strength, indicating that it played a key role as a bridge in the worldwide network of institutional cooperation. Interestingly, the analyses of the co-cited authors showed that Chapple, who obtained the most co-citations, was from the University of Birmingham. Moreover, the top two co-cited references were from Chapple, indicating his core position in this field. The co-cited authors that ranked second and third were Hajishengallis and Halliwell from the University of Pennsylvania (USA) and the National University of Singapore, respectively. Notably, Halliwell was not in the periodontal field, but a researcher in biochemistry. He made pioneering achievements in free radical chemistry, including the roles of free radicals and antioxidants in human diseases (38)

Exploring the mechanism of oxidative stress in periodontitis reflected the integration of different disciplines. Therefore, highly productive and co-cited publications in this field were not limited to periodontal disease-related journals, but some biochemical magazines, such as Free Radical Biology and Medicine, Antioxidants, and Journal of Biological Chemistry, were also included. These journals effectively promoted the integration and development of different subjects.

The analysis of co-occurrence keywords revealed a close relationship between oxidative stress and inflammation in periodontitis; this finding is consistent with the research results in the past few years (8, 39). Periodontitis has been confirmed to be related to the hyperactivity of peripheral blood neutrophils, which are considered the major source of ROS (40). Under a pathological condition, oxidative stress produced by neutrophils leads to the opening of inter-endothelial junctions and promotes the migration of inflammatory cells across the endothelial barrier. Migrating inflammatory cells not only helps clear pathogens and foreign particles but also causes tissue damage (41). Lipid peroxidation and protein and DNA damage are effects that enable ROS to directly induce periodontal tissue damage (42). In addition to the direct effects, oxidative stress also acts as intracellular signaling molecules during osteoclastogenesis, and thus, it can also play an indirect role in bone destruction (5). On the basis of this finding, antioxidants as antagonists of ROS may be an effective strategy for treating chronic periodontitis. Some scholars conducted relevant research to scavenge peroxides in various ways, including the synthesis of nanomaterials or the search for antioxidant components in natural products (43, 44). Studies in this direction will receive continuous attention in the future.

In accordance with the results on keywords co-occurrence, we also found some words related to systemic diseases, such as diabetes, atherosclerosis, and rheumatoid arthritis. These results are also consistent with the research hot spot of periodontitis and systemic diseases in recent years. These complex multifactorial disorders share characteristics with periodontitis, including upregulated production of ROS and accelerating the development of oxidative stress (45). Hajishengallis’s recent research indicated that periodontitis is a dysbiotic inflammatory disease with an adverse effect on systemic health; some studies have also shown that the local treatment of periodontitis reduces systemic inflammation and surrogate markers of comorbid diseases (46, 47). Considerable evidence that demonstrates that periodontitis can be influenced by systemic low-grade inflammation due to different chronic inflammatory conditions is also available (48). Overall, periodontitis and systemic inflammatory diseases exhibit a bidirectional relationship. Oxidative stress is one of the common factors used to explain the pathophysiological mechanism of various inflammatory diseases (49, 50). On this basis, we should comprehensively consider local and systematic factors in diagnosis and treatment, and oxidative stress may also be a key target in the treatment of inflammatory diseases.

By systematically summarizing the literature in this field, this study shows the dynamic development process and structural relationship of relevant scientific knowledge in the form of knowledge map and data visualization, and it explores some frontier hot fields. These explorations will help researchers in this field to conduct in-depth research in clinical and academic fields. It is suggested that in the fields of periodontal disease and oxidative stress, we should pay attention to scientific research hotspots and maintain exchange and cooperation among research institutions to promote the academic development of this discipline. In clinical practice and experimental research, we need to pay more attention towards the mechanism of inflammation and oxidative stress, and further explore the close relationship between reactive oxygen species and periodontitis, as well as the relationship between local and systemic diseases.

## Conclusion

In this study, bibliometrics analysis provided a systematic view of oxidative stress associated with periodontitis. Attention to this field is predicted to continue increasing. Oxidative stress may be a key target in the treatment of inflammatory diseases. The relationship between systemic inflammatory diseases and periodontitis deserves further exploration.

## Limitations

To our knowledge, this work is the first bibliometric analysis of oxidative stress associated with periodontitis; however, some limitations should be addressed. First, the deadline for researched publications was June 11, 2022, but WOSCC also keeps updating. Some new publications will continue to be launched in 2022, and these publications were not included in this work. Second, the WOSCC database was the only source in our study; thus, a few documents from other databases were missed. Third, only articles and reviews written in English were included, and thus, the final results were affected by the search strategy and inclusion/exclusion criteria. However, we believe this work can still be used to present the overall situation and trends in this field. We hope that a more in-depth analysis will contribute to this field in the future.

## Data availability statement

The raw data supporting the conclusions of this article will be made available by the authors, without undue reservation.

## Author contributions

Conceptualization: XRX and WXY. Data collection and analysis: XRX and XCX. Writing—original draft: XRX. Software: XRX and YX. Writing-review and editing: QL, HNM, XCL, and YBH. Funding acquisition: WXY. All authors contributed to the article and approved the submitted version.

## Funding

This work is supported by the Science and Technology Project of Jilin Provincial Department of Finance(Grant Number:JCSZ2021893-22) and International Science and Technology Cooperation Fund(Grant Number:20220402069GH).

## Conflict of interest

The authors declare that the research was conducted in the absence of any commercial or financial relationships that could be construed as a potential conflict of interest.

## Publisher’s note

All claims expressed in this article are solely those of the authors and do not necessarily represent those of their affiliated organizations, or those of the publisher, the editors and the reviewers. Any product that may be evaluated in this article, or claim that may be made by its manufacturer, is not guaranteed or endorsed by the publisher.
